# Application of artificial neural networks to predict the COVID-19 outbreak

**DOI:** 10.1186/s41256-020-00175-y

**Published:** 2020-11-23

**Authors:** Hamid Reza Niazkar, Majid Niazkar

**Affiliations:** 1 Gonabad University of Medical Sciences, Gonabad, Iran; 2 Department of Civil and Environmental Engineering, Shiraz University, Shiraz, Iran

**Keywords:** COVID-19 outbreak, SARS-COV-2, Prediction model, Artificial neural network, Artificial intelligence, Estimation model

## Abstract

**Background:**

Millions of people have been infected worldwide in the COVID-19 pandemic. In this study, we aim to propose fourteen prediction models based on artificial neural networks (ANN) to predict the COVID-19 outbreak for policy makers.

**Methods:**

The ANN-based models were utilized to estimate the confirmed cases of COVID-19 in China, Japan, Singapore, Iran, Italy, South Africa and United States of America. These models exploit historical records of confirmed cases, while their main difference is the number of days that they assume to have impact on the estimation process. The COVID-19 data were divided into a train part and a test part. The former was used to train the ANN models, while the latter was utilized to compare the purposes. The data analysis shows not only significant fluctuations in the daily confirmed cases but also different ranges of total confirmed cases observed in the time interval considered.

**Results:**

Based on the obtained results, the ANN-based model that takes into account the previous 14 days outperforms the other ones. This comparison reveals the importance of considering the maximum incubation period in predicting the COVID-19 outbreak. Comparing the ranges of determination coefficients indicates that the estimated results for Italy are the best one. Moreover, the predicted results for Iran achieved the ranges of [0.09, 0.15] and [0.21, 0.36] for the mean absolute relative errors and normalized root mean square errors, respectively, which were the best ranges obtained for these criteria among different countries.

**Conclusion:**

Based on the achieved results, the ANN-based model that takes into account the previous fourteen days for prediction is suggested to predict daily confirmed cases, particularly in countries that have experienced the first peak of the COVID-19 outbreak. This study has not only proved the applicability of ANN-based model for prediction of the COVID-19 outbreak, but also showed that considering incubation period of SARS-COV-2 in prediction models may generate more accurate estimations.

## Introduction

The novel coronavirus (COVID-19 or SARS-COV-2) epidemic has involved into a global pandemic. More than 17 million individuals have been infected globally, yielding to more than 667,000 death cases from 20 January to 30 July 2020 [1–3]. Rapid human-to-human transmission accompanied by unrevealed nature of the virus has led to a tremendous outbreak. To defeat the COVID-19 outbreak, appropriate and evidence-based actions must be taken worldwide. For this purpose, prediction models can help not only allocating medical resources but also raising the preparedness of healthcare systems involved.

In this regard, mathematical, dynamical and statistical methods have been utilized to forecast the COVID-19 outbreak [4–8]. Some models suggested for this purpose include susceptible-exposed-infectious-recovered (SEIR) model [9], logistic growth model [10] and Adaptive Neuro-fuzzy Inference System (ANFIS) model [11]. For instance, Al-qaness et al. [11] modified ANFIS model using Pollination Algorithm and Salp Swarm Algorithm for prediction of the spreading of COVID-19. Fu et al. [12] utilized a Boltzmann function-based approach for estimation of cumulative confirmed cases in China [12]. Niazkar and Niazkar [13] exploited multi-gen genetic programming, which is one of AI models, to develop mathematical models with the exponential function for predicting the COVID-19 pandemic in seven countries including China, Republic of Korea, Japan, Italy, Singapore, Iran and United States of America. They suggested country-based prediction models. They recommended that the COVID-19 outbreak in each country requires to be investigated separately. Furthermore, Li et al. [14] suggested an exponential function to predict the trend of the COVID-19 outbreak. They estimated the end of the COVID-19 pandemic in China to be after 20 March 2020, while about 52,000 to 68,000 infected and 2400 death cases were predicted [14]. Additionally, Hu et al. [15] proposed a method called modified stacked auto-encoder, which was inspired by an artificial intelligence (AI) model, for real-time forecasting of COVID-19. Also, they predicted the middle of April as the end of the epidemics of COVID-19 [15]. Moreover, Yang et al. [16] developed a SEIR model and AI approach, which was trained by the 2003 SARS data for prediction of COVID-19 in China.

To our knowledge, application of Artificial Neural Networks (ANN) to predict the COVID-19 outbreak is limited. Al-Najjar and Al-Rousan [17] utilized ANN for the prediction of recovered and death cases by using a majority of clinical characteristics, while it may be hard to gather such detailed information required for prediction purposes. Based on the rich and specific input data required by their proposed model, it was found to be satisfactory efficient. Moreover, it will not give a prediction of probable confirmed cases in future. According to the literature, the quest for not only an accurate but also a reliable prediction model of the COVID-19 outbreak is still ongoing. This study aimed to assess the applicability of ANN for predicting daily number of confirmed cases. For this purpose, fourteen ANN-based models were developed to estimate the epidemic outbreak of seven countries (China, Japan, and Singapore, Iran, Italy, South Africa and the United States of America (USA)), together with comparison.

## Methods

### Study design

This study was designed to apply ANN to forecast confirmed cases of the COVID-19 outbreak considering the estimated incubation period of COVID-19, which is known to be between two to fourteen days [18]. In this regard, the total confirmed cases of COVID-19 in China, Japan, and Singapore, Iran, Italy, South Africa and USA were used in this study. Among many countries infected by COVID-19, these countries were selected across different continents and culture centers to reflect diversity. Moreover, their numbers of confirmed cases are order of magnitudes different, which provides an adequate opportunity to test the proposed models for countries with both high and low numbers of confirmed cases. Furthermore, a few of these countries have been infected by SARS-COV-2 for a relatively longer period than many other countries, which is another reason for selecting them. The corresponding data were divided into two parts including train and test data. The former was used to train the ANN-based models, while the latter was exploited for comparison purposes. Thus, the estimated accumulative confirmed cases of the test data were compared with those of observed ones.

### Model development

ANN is a well-documented AI model inspired by the framework of biological human neurons. It has been successfully applied to numerous problems in different fields [19–21]. In essence, it is a powerful tool for finding a relationship between input and output data. For accomplishing this purpose, it is required to be trained using a set of records comprising input and the corresponding output data. The training process is commonly conducted by the flexible architecture of ANN including three layers: (1) an input layer, (2) a hidden layer, and (3) an output layer. The first and the third ones contain neurons associated with the input and output vectors, respectively [22]. On the other hand, neurons in the hidden layer, which are connected with the neurons of the input and output layers, are basically responsible for turning the input data into the corresponding output data. Additionally, they transfer a weighted summation of the input data using a transfer function. Generally, neurons in each layer of ANN are allowed to have connections to those of the next and previous layers, while inter-layered connections are forbidden. The data flow through the network continues until a relation with a desired precision is obtained. Finally, the better ANN is trained, the more accurate results may be achieved [19].

**Fig. 1:**
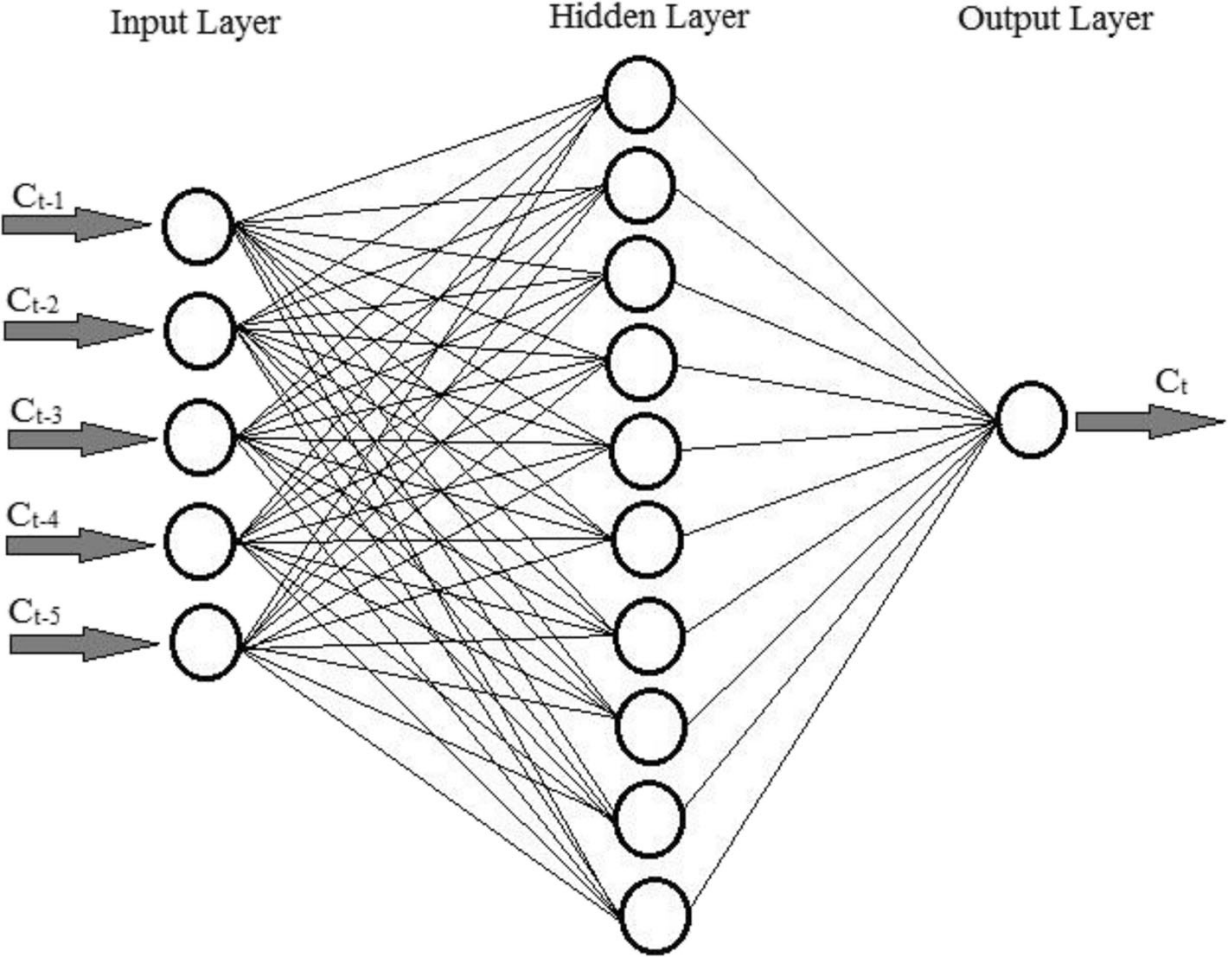
A schematic view of the 5th ANN-based model

In this study, a feed forward back propagation network with the Levenberg Marquardt optimization algorithm were used to train ANN using MATLAB, while common characteristics of ANN were set in accordance with those used in the previous studies [19–21]. In this study, fourteen ANN-based models for estimating daily confirmed cases are proposed. These models are summarized in Table 1. In this table, *f*_*i*_ is the i^th^ function (*i* = 1, 2, …, 14) needed to be approximated by ANN and *C*_*t*_ is number of daily confirmed cases at time *t*. For better clarification, a schematic view of the 5th ANN-based model is shown in Fig. 1.

**Table 1:** Fourteen ANN-based models to predict the COVID-19 outbreak

Models	Equations
1st model	*C*_*t*_ = *f*_1_(*C*_*t* − 1_)
2nd model	*C*_*t*_ = *f*_2_(*C*_*t* − 1_, *C*_*t* − 2_)
3th model	*C*_*t*_ = *f*_3_(*C*_*t* − 1_, *C*_*t* − 2_, *C*_*t* − 3_)
4th model	*C*_*t*_ = *f*_4_(*C*_*t* − 1_, *C*_*t* − 2_, *C*_*t* − 3_, *C*_*t* − 4_)
5th model	*C*_*t*_ = *f*_5_(*C*_*t* − 1_, *C*_*t* − 2_, *C*_*t* − 3_, *C*_*t* − 4_, *C*_*t* − 5_)
6th model	*C*_*t*_ = *f*_6_(*C*_*t* − 1_, *C*_*t* − 2_, *C*_*t* − 3_, *C*_*t* − 4_, *C*_*t* − 5_, *C*_*t* − 6_)
7th model	*C*_*t*_ = *f*_7_(*C*_*t* − 1_, *C*_*t* − 2_, *C*_*t* − 3_, *C*_*t* − 4_, *C*_*t* − 5_, *C*_*t* − 6_, *C*_*t* − 7_)
8th model	*C*_*t*_ = *f*_8_(*C*_*t* − 1_, *C*_*t* − 2_, *C*_*t* − 3_, *C*_*t* − 4_, *C*_*t* − 5_, *C*_*t* − 6_, *C*_*t* − 7_, *C*_*t* − 8_)
9th model	*C*_*t*_ = *f*_9_(*C*_*t* − 1_, *C*_*t* − 2_, *C*_*t* − 3_, *C*_*t* − 4_, *C*_*t* − 5_, *C*_*t* − 6_, *C*_*t* − 7_, *C*_*t* − 8_, *C*_*t* − 9_)
10th model	*C*_*t*_ = *f*_10_(*C*_*t* − 1_, *C*_*t* − 2_, *C*_*t* − 3_, *C*_*t* − 4_, *C*_*t* − 5_, *C*_*t* − 6_, *C*_*t* − 7_, *C*_*t* − 8_, *C*_*t* − 9_, *C*_*t* − 10_)
11th model	*C*_*t*_ = *f*_11_(*C*_*t* − 1_, *C*_*t* − 2_, *C*_*t* − 3_, *C*_*t* − 4_, *C*_*t* − 5_, *C*_*t* − 6_, *C*_*t* − 7_, *C*_*t* − 8_, *C*_*t* − 9_, *C*_*t* − 10_, *C*_*t* − 11_)
12th model	*C*_*t*_ = *f*_12_(*C*_*t* − 1_, *C*_*t* − 2_, *C*_*t* − 3_, *C*_*t* − 4_, *C*_*t* − 5_, *C*_*t* − 6_, *C*_*t* − 7_, *C*_*t* − 8_, *C*_*t* − 9_, *C*_*t* − 10_, *C*_*t* − 11_, *C*_*t* − 12_)
13th model	*C*_*t*_ = *f*_13_(*C*_*t* − 1_, *C*_*t* − 2_, *C*_*t* − 3_, *C*_*t* − 4_, *C*_*t* − 5_, *C*_*t* − 6_, *C*_*t* − 7_, *C*_*t* − 8_, *C*_*t* − 9_, *C*_*t* − 10_, *C*_*t* − 11_, *C*_*t* − 12_, *C*_*t* − 13_)
14th model	*C*_*t*_ = *f*_14_(*C*_*t* − 1_, *C*_*t* − 2_, *C*_*t* − 3_, *C*_*t* − 4_, *C*_*t* − 5_, *C*_*t* − 6_, *C*_*t* − 7_, *C*_*t* − 8_, *C*_*t* − 9_, *C*_*t* − 10_, *C*_*t* − 11_, *C*_*t* − 12_, *C*_*t* − 13_, *C*_*t* − 14_)

According to models presented in Table 1, when *f*_*i*_ is found, it can be used for future prediction of the COVID-19 outbreak. The architecture of ANN used to find *f*_*i*_ has one, ten and one neurons in the input, hidden, and output layers, respectively. Since the 14th model introduced in Table 1 uses the daily confirmed cases of the fourteen previous days, the first fourteen days of data were excluded from the whole data to enable training of all ANN-based models. Thus, the adopted data of the COVID-19 outbreak were divided into two parts: (1) train data (50 days of data) and (2) test data (13 days of data). The former was used to train ANN, while the latter was utilized to evaluate the performance of ANN-based models in predicting the COVID-19 outbreak. The immediate results achieved by ANN were rounded up to integer values using the round function embedded in Excel [23].

### Data sources

Chronological data of confirmed and death cases of SARS-COV-2 were gathered from World Health Organization (WHO) situation reports [1] and the National Health Commission of the People’s Republic of China (NHC) official website [24]. The latter were preferred for China when there was a discrepancy between those two data sources. From 17 February 2020, both laboratory-confirmed and clinically-diagnosed cases were reported in China, which led to a rapid rise in the corresponding data. In this study, confirmed cases of the Republic of China, Japan, Singapore, Iran, Italy, South Africa and USA were considered.

### Data analysis

**Fig. 2:**
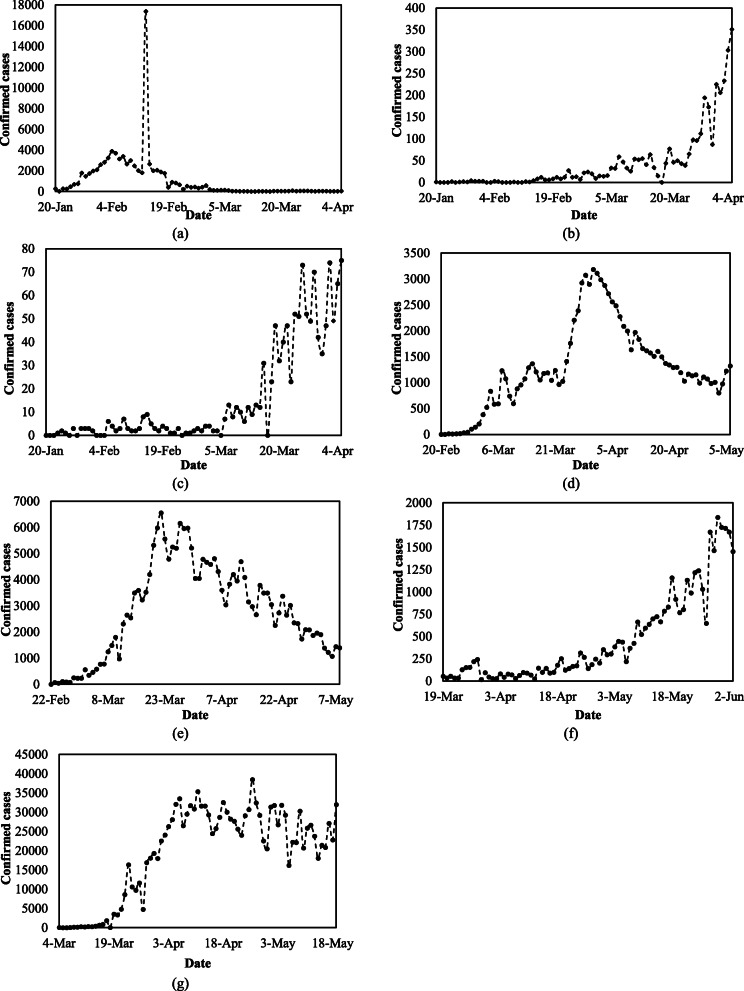
Daily confirmed cases of COVID-19 in (**a**) China, (**b**) Japan, (**c**) Singapore, (**d**) Iran, (**e**) Italy, (**f**) South Africa, and (**g**) USA

Descriptive analysis of the gathered data was conducted using Excel, which offers robust facilities for analyzing data and implementing numerical methods [23]. The results of this analysis and the period of data used for each country are summarized in Table 2. As shown, the data of China and USA have a wider range than that of others based on maximum, minimum values and standard deviation. Furthermore, Fig. 2 depicts that the data period of each country is different, while the data of each country commonly starts as the first positive cases were reported. In addition, it manifests significant fluctuations in the daily confirmed cases of the seven countries considered. The wide range and considerable variations of data demand a powerful tool to predict the COVID-19 outbreak.

**Table 2:** Descriptive Analysis of the confirmed cases of COVID-19 in China, Japan, Singapore, Iran, Italy, South Africa and USA

Country	Data period in year 2020	Min	Max	Mean	Median	Standard deviation	Confidence Level (95.0%)
China	20 January to 5 April	8	17,382	1063	259	2187	496
Japan	20 January to 5 April	0	71	42	13	71	16
Singapore	20 January to 5 April	0	75	15	4	22	5
Iran	20 February to 6 May	2	3186	1298	1192	96	190
Italy	22 February to 8 May	6	6557	2803	2729	206	409
South Africa	19 March to 3 June	17	1837	464	243	57	114
USA	22 February to 8 May	19	38,509	19,421	22,813	1369	2726

Three dimensionless criteria were selected from the literature to evaluate the performance of ANN-based models in predicting the COVID-19 outbreak [19, 25, 26]: (1) Normalized Root Mean Square Error (NRMSE), (2) Mean Absolute Relative Error (MARE), and (3) determination coefficient (R^2^). These indices are written for daily confirmed cases in Eq. 1 to Eq. 3, respectively:

1 $$ \mathrm{NRMSE}=\frac{\sqrt{\frac{1}{N}\sum \limits_{\mathrm{i}=1}^{\mathrm{N}}{\left[{C}_{i, observed}-{C}_{i, estimated}\right]}^2}}{{\mathrm{C}}_{\mathrm{observed},\max }-{\mathrm{C}}_{\mathrm{observed},\min }} $$

2 $$ \mathrm{MARE}=\frac{1}{N}\sum \limits_{i=1}^N\mid \frac{C_{i, observed}-{C}_{i, estimated}}{C_{i, observed}}\mid \times 100 $$

3 $$ {\mathrm{R}}^2={\left(\frac{\sum \limits_{\mathrm{i}=1}^{\mathrm{N}}\left[\left({C}_{i, observed}-\frac{\sum \limits_{i=1}^N{C}_{i, observed}}{N}\right)\left({C}_{i, estimated}-\frac{\sum \limits_{i=1}^N{C}_{i, estimated}}{N}\right)\right]}{\sqrt{\sum \limits_{\mathrm{i}=1}^{\mathrm{N}}\left[{\left({C}_{i, observed}-\frac{\sum \limits_{i=1}^N{C}_{i, observed}}{N}\right)}^2{\left({C}_{i, estimated}-\frac{\sum \limits_{i=1}^N{C}_{i, estimated}}{N}\right)}^2\right]}}\right)}^2 $$

where *C*_*i*, *observed*_ and *C*_*i*, *estimated*_ are the *i*^*th*^ observed and estimated values of daily confirmed cases, respectively, C_observed, max_ and C_observed, min_ are the maximum and minimum numbers of daily confirmed cases, respectively, *i* is the counter, and *N* is the total number of data. According to Eq. 1 to Eq. 3, a prediction model performs more accurately when it yields to lower values of NRSME and MARE and higher R^2^ values.

## Results

**Fig. 3:**
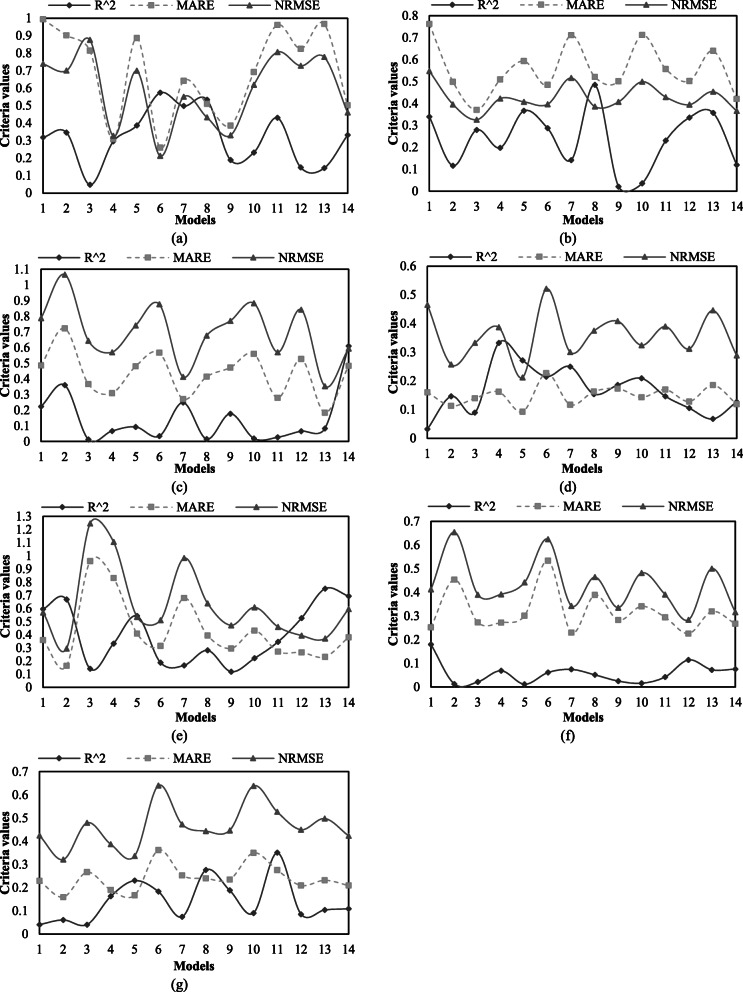
Comparison of different ANN-based models using R^2^, MARE and NRMSE for (**a**) China, (**b**) Japan, (**c**) Singapore, (**d**) Iran, (**e**) Italy, (**f**) South Africa, and (**g**) USA

After training ANN, the proposed models were used to predict the daily confirmed cases of COVID-19 in each country. Figure 3 illustrates the values of R^2^, MARE and NRMSE obtained by the ANN-based models in predicting the COVID-19 outbreak in the seven countries considered. As shown, the proposed ANN-based models have different performances. Additionally, applying only confirmed cases of the very previous day by ANN for estimation daily confirmed cases (the 1st model) do not yield to reliable predictions for seven considered countries.

In order to determine the three best ANN-based models, a ranking system with equal weight for each accuracy index, which was adopted from the literature [27], was used to rate each and every ANN-based model. This system identifies models with the best and worst performances. Based on this system, each ANN-based model is first ranked in accordance with their values obtained for MARE, NRMSE and R^2^. Afterwards, the algebraic summation of the three ranking values was computed and used as a new reference for ranking the performance of each ANN-based model for each country. The final results of applying this ranking system are presented in Table 3 for China, Japan, Singapore, Iran, Italy, South Africa, USA and overall. The lower the rank of a model is, the more accurate it performs.

**Table 3:** Ranking the ANN-based models for estimating confirmed cases of the test data

Models	Ranks							
	China	Japan	Singapore	Iran	Italy	South Africa	USA	Overall
1st model	12	12	8	13	5	4	8	11
2nd model	9	7	11	3	1	14	3	5
3th model	13	1	9	8	14	7	13	12
4th model	3	8	4	6	12	5	2	4
5th model	7	6	7	1	7	11	1	2
6th model	1	3	13	12	8	12	12	13
7th model	4	13	1	2	13	3	11	6
8th model	2	2	10	8	10	10	5	8
9th model	6	10	6	10	9	6	6	9
10th model	8	14	14	5	11	12	13	14
11th model	10	11	5	11	4	8	9	10
12th model	11	3	12	7	3	1	7	2
13th model	14	8	2	14	1	9	10	7
14th model	5	3	3	4	5	2	4	1

**Fig. 4:**
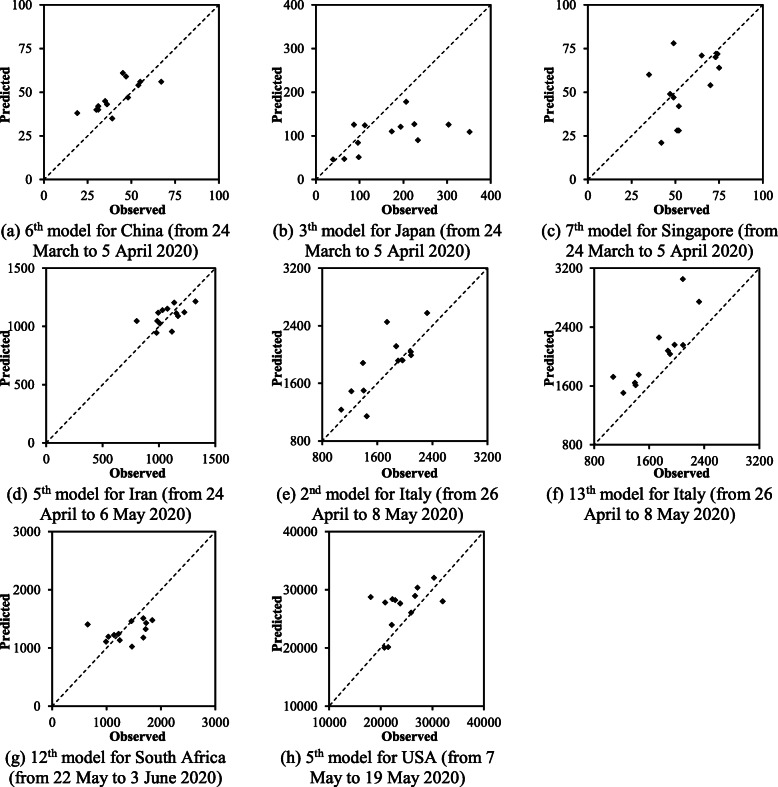
Predicted versus observed daily confirmed cases of COVID-19 in different countries

Based on the ranking results shown in Table 3, the ANN-based models with the best rank were selected, and the corresponding predictions are compared with the observed ones in Fig. 4 for the test data in different countries. Each plot in Fig. 4 depicts the observed versus predicted values of daily confirmed cases. In other words, the abscissa and ordinate of each point shown in Fig. 4 represent the observed and predicted confirmed case for the same date, respectively. In this regard, the closer the point to the identity line, the closer the predicted value to the observed one. Also, the further one point locates from the line of equality; the less accurate the number of confirmed cases is estimated. The details of the achieved results are presented separately for each country in the following:

- Estimating the COVID-19 outbreak in China

The performances of the ANN-based models for China from 24 March to 5 April 2020 are shown in Fig. 3a. Based on Fig. 3a and Table 3, the 6th, 8th, 4th, 7th, and 14th models achieve closer confirmed cases to the observed values of China in comparison with other ANN-based models. Although prediction results of any forecasting models are accompanied by marginal errors, the mentioned ANN-based models forecast number of daily confirmed cases of COVID-19 close to the observed ones. These precise estimations are promising considering the fact that the temporal variations of daily confirmed cases depicted in Fig. 2 demonstrate significant fluctuations. Moreover, the period of estimation of confirmed cases in China is after the first peak of the COVID-19 outbreak. This helps ANN-based models to perform adequately as it provides the chance of being familiar with the trend of the outbreak in the train data. Consequently, the mentioned ANN-based models are suggested to predict confirmed cases when the first peak of the COVID-19 outbreak is experienced within the train data.

b Estimating the COVID-19 outbreak in Japan

Fig. 4b compares the best estimated numbers of confirmed cases of Japan with the corresponding observed ones. As shown, most of the points in Fig. 4b are beneath the line of equality. This indicates that the ANN-based models overall underestimated the daily confirmed cases of COVID-19 in Japan during 24 March to 5 April 2020, particularly when numbers of daily confirmed cases are high. This may be due to the sharp rise of confirmed cases observed in Japan in this time interval, which is associated with the first peak of the confirmed cases in this country. In other words, the confirmed cases from 3 February to 23 March 2020, which were used to train ANN models, are lower than those in 24 March to 5 April 2020 (test data). To be more specific, ANN did not train with a good set of data and consequently, the ANN-based models did not perform as adequate as expected. The discrepancy between the observed and predicted number of confirmed cases increases when high records of confirmed cases are observed. Thus, the proposed ANN-based models may perform less precisely when they are used before the first peak of the COVID-19 outbreak. According to Fig. 3b and Table 3, the 3th, 8th, 14th and 12th models perform slightly better than the other ANN-based models. Finally, the uncertainty in a long-term estimation using any prediction model is significantly higher than that of a short-term forecasting, particularly when a country has not experienced the rising limb of the outbreak.

c Estimating the COVID-19 outbreak in Singapore

The performance of the ANN-based models in predicting daily confirmed cases in Singapore is illustrated in Fig. 3c for the test data. As shown in Table 3 and Fig. 3c, the 7th, 13th and 14th ANN-based models estimated the confirmed cases with a very high accuracy in comparison with other ones. Based on Fig. 2, the maximum numbers of daily confirmed cases in Singapore are as high in the train data as those in the test data. This mainly provides ANN with a sufficient train data so that several ANN-based models can predict satisfactory estimations. Consequently, Fig. 3a and Fig. 3c schematically exhibit the acceptable performance of several ANN-based models in predicting the confirmed cases from 24 March to 5 April 2020 in China and Singapore, respectively.

d Estimating the COVID-19 outbreak in Iran

Iran is one of the earliest countries to severely suffer from COVID-19. Figure 3d depicts the performance of the ANN-based models in predicting positive cases of COVID-19 from 24 April to 6 May 2020. The obtained precision may be because of the time interval of Iran’s COVID-19 data considered in this study, which includes both the rising and falling limbs of the first outbreak peak in this country, as shown in Fig. 2. Among the ANN-based models shown in Table 3 and Fig. 3d, the 5th, 7th, 2nd and 14th models performed as the first four best models because they reach the lowest NRMSE and MARE and highest R^2^.

e Estimating the COVID-19 outbreak in Italy

Italy has experienced a relatively considerable positive confirmed and death cases of COVID-19 among European countries. The performance of different ANN-based models for predicting confirmed cases of Italy are compared in Fig. 3e. Based on the results, the maximum limit of the predicted cases varies between 3200 and 4000 positive cases. This reveals a discrepancy between the performances of different ANN-based models for Italy’s data. According to Table 3 and Fig. 3e, the 2nd, 13th, 12th, 14th and 1st models reached better estimations in comparison with other models.

f Estimating the COVID-19 outbreak in South Africa

Among different ANN-based models used in predicting the COVID-19 outbreak in South Africa, the 12th, 14th and 7th models have the best performance based on Table 3 and Fig. 3f. According to Fig. 2, the data period used for South Africa indicates that the general trend of the data is monotonically increasing. This implies the maximum of the train data was lower than the maximum of the test data. This may yield to relatively lower values of R^2^ and relatively higher values of NRMSE and MARE comparing with those achieved for other countries.

g Estimating the COVID-19 outbreak in USA

The COVID-19 data of USA reported by WHO was zero for the 8th, 9th, 15th, 16th and 22th March 2020 and negative for the 10th May 2020. These values were substituted by 144, 148, 672, 872, 8631and 20,697, respectively, while they were adopted from Wikipedia online resources [28]. The criteria values for the predicted cases of COVID-19 from 7 May to 19 May 2020 are presented in Fig. 3g. As depicted in Table 3 and Fig. 3g, the 5th, 4th, 2nd and 14th models perform better than other models. Finally, Fig. 3g demonstrates that several ANN-based models are capable of predicting the COVID-19 outbreak particularly when a large number of cases need to be estimated.

## Discussion

As previously mentioned, this study has exploited ANN to forecast the daily confirmed cases of the COVID-19 outbreak. First of all, ANN generally estimates the best when it is used for prediction within the range of trained data. In particular, prediction of future number of confirmed cases inevitably contains time values out of the range of the train data. To be more precise, such application of ANN may be suitable for short-term estimation such as several days ahead. However, short-term predictions do not provide an adequate perspective of the COVID-19 outbreak for healthcare decision makers. Also, ANN can be used as a prediction tool for time series data, while such applications require the presence of a large database so that ANN can capture possible patterns. However, such database is not available for the COVID-19 outbreak in this time interval. These shortcomings may confine the use of ANN for predicting the COVID-19 outbreak, while this study proposed fourteen novel ANN-based models for this purpose.

After applying fourteen ANN-based models to predict the COVID-19 outbreak in seven countries, the values of NRMSE, MARE and R^2^ were used to calculate the minimum, average and maximum of the three metrics for all models in each country. Comparison of the ranges of R^2^ shown in Fig. 3 demonstrates that the average R^2^ of the ANN-based models for Italy, Singapore and China are not only more than 0.5 but also better than those of others, while the lowest R^2^ values were obtained for South Africa. Additionally, Fig. 3 depicts that the best average MARE and NRMSE values are achieved for Iran, whereas the highest average MARE and NRMSE values were obtained for China and Italy, respectively. The results also show that Italy has the best average R^2^ and the highest NRMSE. This clearly indicates considering only one metric may not be enough to assess the performance of prediction results, while several metrics may give a better perspective of the accuracy of the results. According to Fig. 3, the overall performance of ANN-based models for China, Iran and Italy, which include the rising and falling limbs of the first peak, are better than Japan, Singapore, South Africa and USA, which have only a rising limb as shown in Fig. 2. This is mainly because the data by which ANN models were trained contain the maximum confirmed cases, which enables them for more suitable predictions.

According to Table 3, the 14th model achieved the first places in the ranking system, while the 5th and 12st models are overall ranked as the second best models. The 14th model uses the records of previous 14 days for estimation of confirmed cases in each day, while the achieved results reveal that the best predictions can be made using this model. This also equals to the maximum estimated incubation period of SAR-COV-2 [18], which indicates that implementation of incubation period into the prediction model may bring about the best estimations. Finally, the 14th model is suggested to predict daily confirmed cases of COVID-19, particularly for countries that have experienced the first peak of the outbreak.

Application of ANN-based models to predict the COVID-19 pandemic indicates that the best estimation results were not achieved by the same model for all countries considered. Based on Table 3, ANN-based models have different performances and subsequently ranking numbers for different countries. This may be due to the fact that the historical record of the COVID-19 outbreak in each country is somehow unique, as shown in Fig. 2, and different trends of outbreak inevitably demand different prediction models. Therefore, an investigation similar to the one conducted in this study is suggested in favor of finding the best ANN-based model for a specific country, while the 14th model is found to be adequate in the absence of such analysis. The need for the quest for a country-based prediction model is in agreement with the previous studies [5, 13].

The prediction of COVID-19 confirmed cases can provide an approximate number of not only patients expected but also facilities required. Obviously, the more accurate the confirmed cases are estimated, the better perspective of future can be provided. Despite numerous advantages of prediction models, they have inevitable limitations. Since these predictions are based on the reported data, they underestimate the confirmed cases either due to the neglecting asymptomatic patients or limited sources of case findings in some countries [13]. Moreover, developing an ANN-based prediction model, which considers previous fourteen days, reduces fourteen-day records from ANN train data. However, the longer a country has experienced COVID-19, the more number of data is available for the country. This obviously provides more data to train AI models, such as ANN, which may yield to more accurate prediction results [13].

## Conclusions

In this study, fourteen ANN-based models were proposed and compared in a bid to develop the most accurate ANN-based model for estimating the COVID-19 outbreak. The results indicate that implementation of the incubation period of COVID-19 in the ANN-based prediction models led to more accurate estimations. The proposed ANN-based model considering 14-day previous records is suggested to predict approximate numbers of daily confirmed cases, particularly in countries that have experienced the first peak of the COVID-19 outbreak.

## Data Availability

Data and materials involved in this paper are from WHO and NHC, and they are all available online.

## Abbreviations

COVID-19: Coronavirus Disease 2019
ANN: Artificial Neural Networks
SARS-COV-2: Severe Acute Respiratory Syndrome Coronavirus 2
AI: Artificial Intelligence
SEIR: susceptible-exposed-infectious-recovered
ANFIS: Adaptive Neuro-fuzzy Inference System
WHO: World Health Organization
NHC: National Health Commission of the People’s Republic of China
NRMSE: Normalized Root Mean Square Error
MARE: Mean Absolute Relative Error
